# ITSUS: Integrated, Tiered, Self-Directed Ultrasound Scanning for Learning Anatomy

**DOI:** 10.7759/cureus.16119

**Published:** 2021-07-02

**Authors:** Creagh Boulger, Michael Prats, Adam Niku, Martina Diaz, David P Bahner

**Affiliations:** 1 Emergency Medicine, The Ohio State University College of Medicine, Columbus, USA; 2 Cardology, The University of Texas Health Science Center at Houston, Houston, USA; 3 Emergency Medicine, University of Cincinnati Medical Center, Columbus, USA; 4 Emergency Medicine, The Ohio State University Wexner Medical Center, Columbus, USA

**Keywords:** ultrasound, medical student education, independent learning, anatomy, curricular development

## Abstract

Ultrasound is being introduced into many medical schools and incorporated into the anatomy curriculum; however, in most cases, this consists of proctored sessions which can be limited by faculty time and availability. Additionally, the severe acute respiratory syndrome coronavirus-2 (SARS-CoV-2) pandemic has significantly impacted medical education, especially ultrasound education, which has traditionally depended on hands-on practice and instruction. A structured, independent, hands-on learning curriculum using ultrasound would have many benefits. In this study, eight self-guided system-based modules were developed mirroring the undergraduate anatomy curriculum. For each scan, a beginner, intermediate, and advanced component was designed. Each module contains clear, stepwise directions for image acquisition, optimization, and interpretation of the anatomical structures and suggestions for troubleshooting. Students save ultrasound images as part of their digital portfolios for review with ultrasound faculty. This design provides an educational model to increase medical student opportunities for independent, structured, self-directed anatomy learning with ultrasound that can be integrated with existing educational programs.

## Introduction

Ultrasound is a valuable tool for teaching anatomy to medical students, either as a supplement or a replacement for traditional approaches. Several studies showed that ultrasound could increase learning and satisfaction with anatomy curricula by allowing students to visualize anatomical structures in live human models [1-5]. This approach exposes students to point-of-care ultrasound, a skill that is now utilized in several medical specialties [6]. Point-of-care ultrasound is also becoming a required component of many graduate medical education programs. Therefore, early exposure to this skill set will better prepare students for future training. Nevertheless, ultrasound has not been widely integrated into undergraduate medical education (UME) [7].

Although the American Medical Association supports bedside ultrasound using appropriately trained physicians, no organization offers guidelines or standards for ultrasound education at the medical school level [8]. Many barriers exist in utilizing ultrasound for an anatomy training curriculum. There are several barriers to utilizing ultrasound in the anatomy training curriculum. These include, but are not limited to, a lack of standards, a lack of time in existing curricula, the cost of resources, and scarcity of faculty trained in ultrasound [9]. A growing body of literature suggests that perceived confidence in the use of ultrasound is low among graduate-level trainees and correlates with poor objective competency; therefore, it is essential to address these barriers to formal ultrasound training in medical school [9,10].

Furthermore, the severe acute respiratory syndrome coronavirus-2 (SARS-CoV-2) pandemic greatly impacted medical education, especially ultrasound education, which has traditionally depended on hands-on practice and instruction. Evidence suggests that medical school training may positively impact graduates' ultrasound use in subsequent practice [11]. At the UME level, ultrasound in anatomy education traditionally relied on structured teaching by faculty proctors trained in ultrasound. Proctors provide lecture-based education, often complemented by hands-on simulations in which students practice acquisition skills under the supervision of a trained proctor [12-15]. Self-directed, hands-on training has been explored as an alternative approach to teaching basic ultrasound skills. Studies showed that self-directed training curricula could improve self-reported competence and objective assessment scores from baseline while requiring fewer resources to implement [5].

At the Ohio State University College of Medicine (OSUCOM), an integrated UME ultrasound curriculum relies on classroom lectures and supervised hands-on sessions [15]. However, student demand has begun to outpace faculty resources. A self-directed learning (SDL) model was developed combined with the existing curriculum to address this need. This integrated, tiered, self-directed ultrasound (ITSUS) program provides a model for incorporating SDL modalities to enhance and reinforce the UME ultrasound curricula. In resource-restricted settings, learners could use this hands-on curriculum to supplement other asynchronous resources.

## Technical report

UME at OSUCOM is organized according to a “Lead.Serve.Inspire. (LSI)” curriculum. LSI is divided into three sections. Part 1 encompasses years 1 and 2 of medical school and includes preclinical education. Part 2 encompasses year 3 and serves as an introduction to clinical medicine. Part 3 encompasses year 4 and focuses on advanced clinical skills.

LSI Part 1 integrates gross anatomy into a systems-based curriculum. Anatomy laboratories are integrated over the first two years of medical education, as students concurrently learn physiology, pathology, and pharmacology pertaining to each organ system. Part 1 is organized into the following systems-based unit: Foundations, Musculoskeletal, Neurological, Cardiopulmonary, Gastrointestinal/Renal, Endocrine/Reproductive, and Host Defense. All units except Foundations and Host Defense contain a gross anatomy component.

Throughout LSI Part 1, students are offered the opportunity to participate in elective and required hands-on ultrasound scanning sessions relevant to the five curricular units that incorporate a gross anatomy component. These five blocks are divided into six ultrasound sessions: Musculoskeletal, Head and Neck, Cardiac, Hepatobiliary, Renal, and Thyroid. Students can attend one or multiple hands-on sessions covering the sonographic anatomy and physiology relevant to the block they are currently studying. Each session begins with a short lecture covering the basics of ultrasound and indications, acquisition, interpretation, and medical decision-making concerning the pertinent scans for the covered anatomic area. Students then form into small groups and practice scanning in the presence of a trained proctor, often a senior student or resident physician, with a faculty overseeing the session.

**Figure 1:**
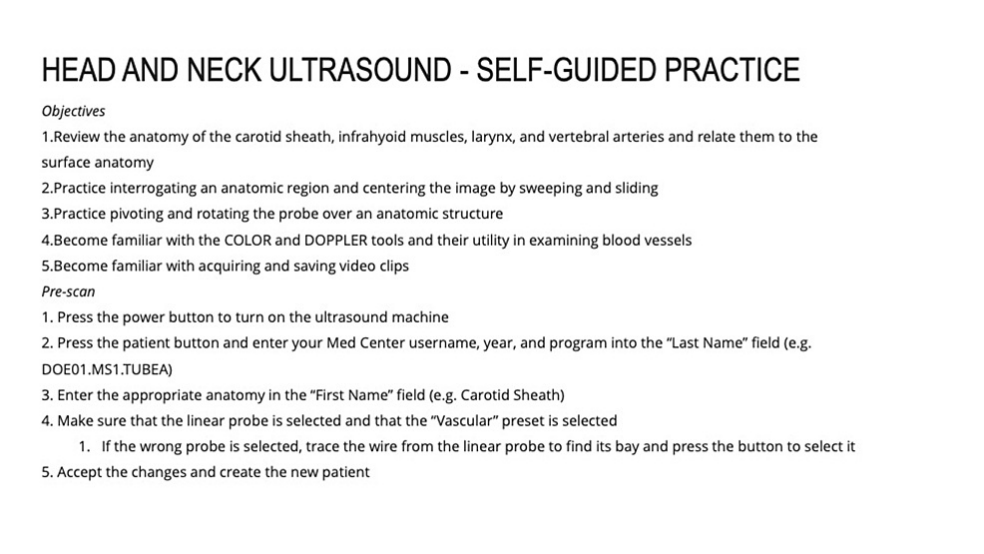
Integrated, Tiered, Self-directed Ultrasound Scanning module example. Head and Neck Module Introduction

**Figure 2:**
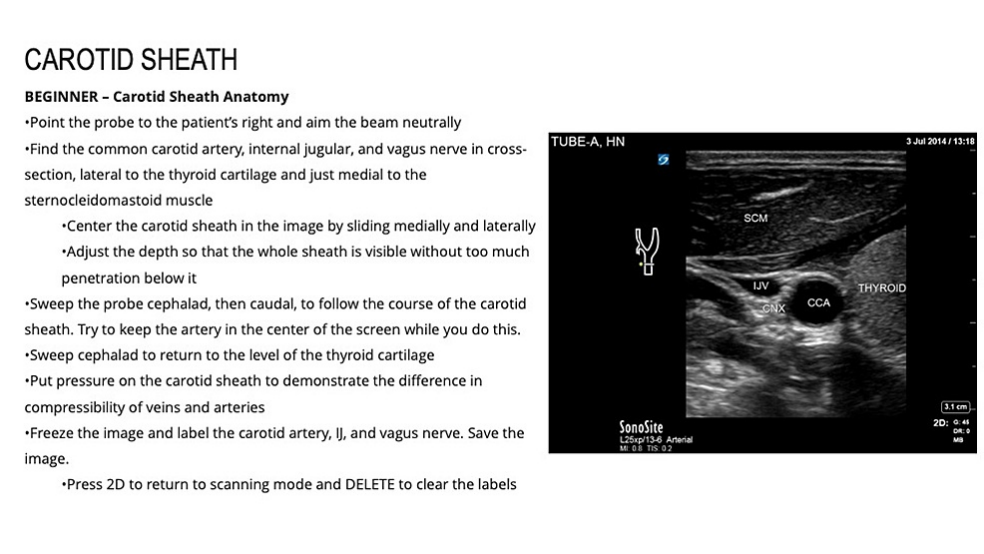
Integrated, Tiered, Self-directed Ultrasound Scanning module example. Head and Neck Module Beginner: Carotid Sheath

**Figure 3:**
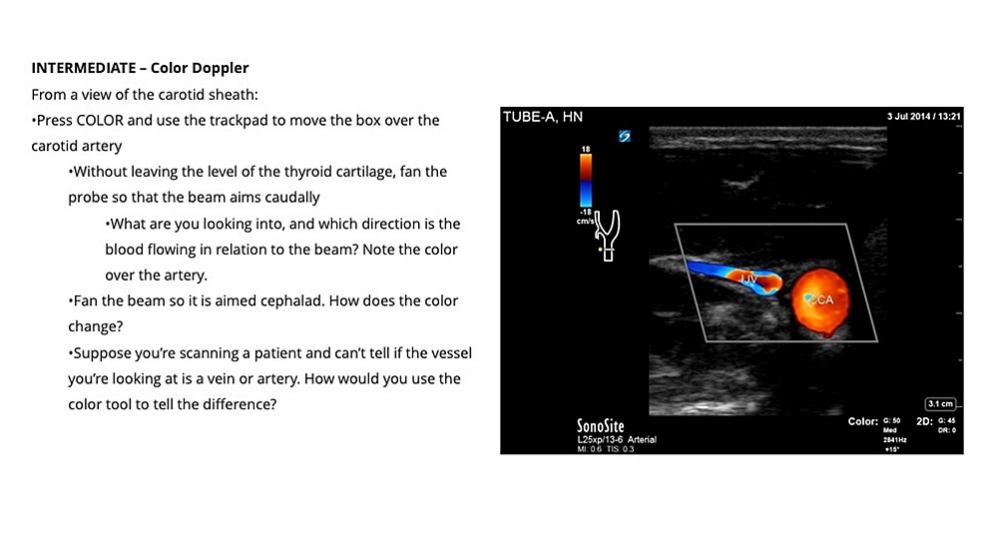
Integrated, Tiered, Self-directed Ultrasound Scanning module example. Head and Neck Module Intermediate: Color Doppler

**Figure 4:**
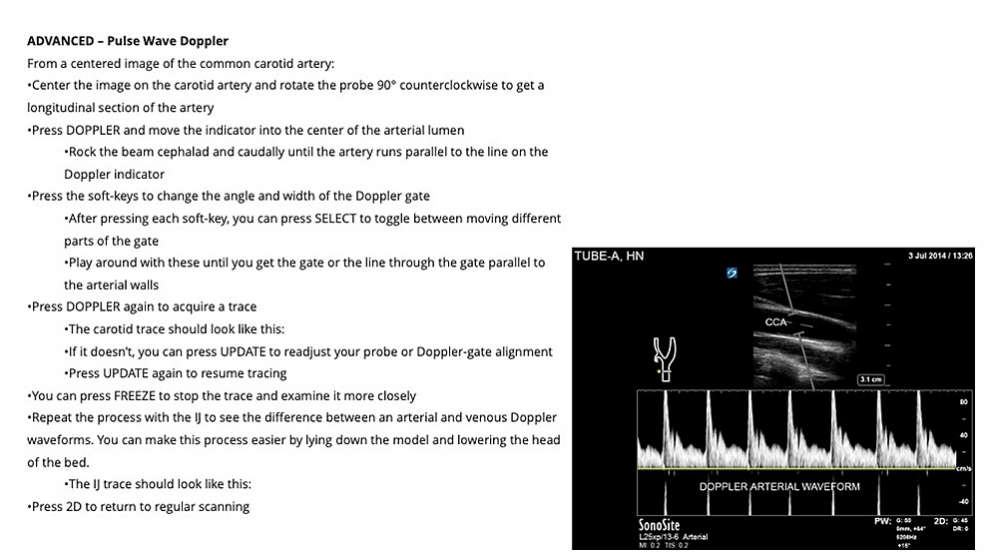
Integrated, Tiered, Self-directed Ultrasound Scanning module example. Head and Neck Module Advanced: Pulse Wave Doppler

**Figure 5:**
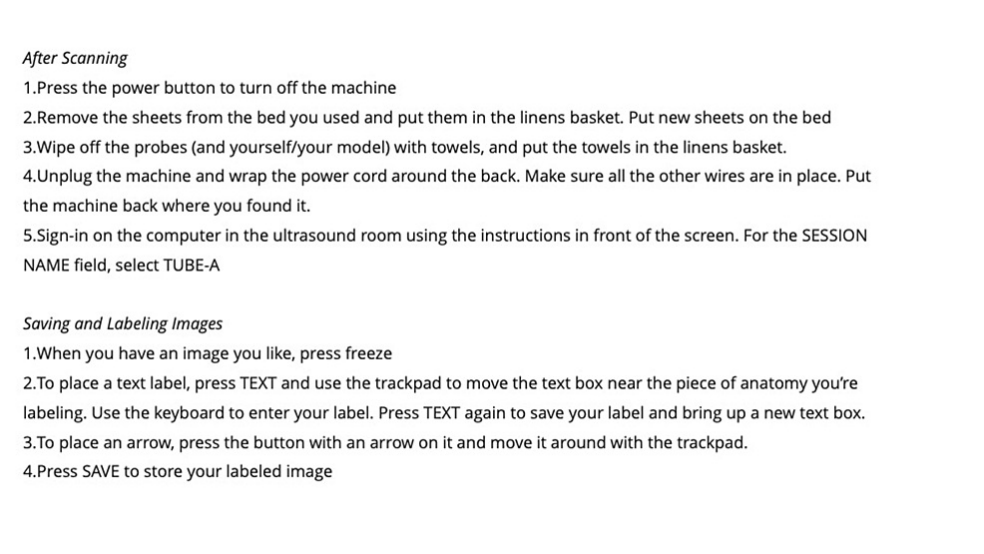
Integrated, Tiered, Self-directed Ultrasound Scanning module example. Head and Neck Module Saving Process

 A series of eight self-directed, hands-on ultrasound modules were designed to correspond to the systems-based anatomy curriculum in LSI Part 1. These eight modules constitute the ITSUS curriculum that supplements the existing formal UME ultrasound education curriculum at OSUCOM. Therefore, students attended their regularly scheduled instruction but were also offered these modules for additional educational opportunities. The modules cover the six previously mentioned ultrasound sessions and provide two additional modules on pelvic anatomy and aortic anatomy. The modules were formatted as web-based electronic worksheets made available for students during independent scanning time or as an adjunct to formal, organized scanning activities (Figures 1-5).

Each module contains clear, stepwise directions for the acquisition, optimization, and interpretation of images. Users are guided through each scan with precise prescriptive language based on the five cardinal probe motions (rock, slide, sweep, fan, and rotate) as taught at OSUCOM [16]. Instructions describe initial probe placement, stepwise transducer movements to interrogate anatomy, and techniques for image optimization. The instructions also include directions for the use of scan-specific knobology and ultrasound images for reference.

Each module is divided into three “tiers”-beginner, intermediate, and advanced. Beginner tasks focus solely on image acquisition and recognition of basic anatomy. Several beginner-level tasks repeat and reinforce scans and techniques covered in the hands-on scanning sessions offered through LSI Part 1, as described above. Intermediate and advanced tasks introduce higher-level objectives that focus on obtaining images of more challenging anatomy and utilize advanced functions and calculations (e.g., measuring E-point septal separation of the anterior leaflet of the mitral valve). Intermediate and advanced tasks are designed to teach students to use ultrasound to examine the physiology and recognize pathology.

Individual ITSUS modules include labelling, saving, and downloading images to a personal ultrasound portfolio (Figure 5). Student portfolios are reviewed periodically with trained ultrasound faculty for quality assurance and formal teaching purposes. Completion of a documented ITSUS portfolio counts toward an Advanced Competency in Ultrasound granted during LSI Part 3.

## Discussion

Ultrasound education is a developing component of medical education and has been a valuable adjunct to traditional anatomy curricula. Despite growing recognition of this importance, fewer than one-third of medical schools currently offer mandatory or elective ultrasounds in their curricula [7,9]. This disparity is likely due to several barriers. Demand for ultrasound training often exceeds the supply of trained proctors, while synchronous educational modalities can reduce individual learners’ flexibility and access to experiential learning.

Ultrasound use in an anatomy curriculum requires both a structured curriculum and access to resources for hands-on practice. Traditionally, this is accomplished by exposing learners to didactic instruction followed by proctored hands-on sessions. Structured, self-directed ultrasound education preserves some of these didactic benefits by substituting the trained proctor with detailed, prescriptive instructions for image acquisition and interpretation. In this way, self-directed scanning represents a unique opportunity to either supplement traditional lecture-based educational modalities or potentially replace some aspects of the traditional integrated UME anatomy ultrasound curriculum. The ITSUS model provides an educational framework to increase medical student opportunities for independent, structured, self-directed ultrasound anatomy learning while addressing many barriers.

The ITSUS curriculum is accessible to students via publication in a web-based format, increasing access to experiential learning. Whereas access to hands-on experience in traditional ultrasound curricula is limited by proctor availability and top-down scheduling restrictions, any learner with access to an ultrasound machine can use this resource to practice acquisition and interpretation skills. The flexibility of the ITSUS curriculum maximizes the potential to increase access to structured anatomy learning and ease demands on trained faculty and proctors.

The tiered approach to task assignment is a crucial innovation as well. A difficulty often encountered by ultrasound educators is simultaneously teaching ultrasound to learners of various interests, experiences, and skill levels. By designating tasks as Basic, Intermediate, and Advanced, ITSUS is appropriate for learners at all levels of medical school education and allows learners to advance at their own pace. Beginner ultrasound learners are free to focus on basic tasks that teach fundamental B-mode acquisition and interpretation. More advanced learners can bypass the basic level tasks to focus on advanced techniques to demonstrate dynamic physiology. Students can benefit from repeating modules as they develop their skills, completing progressively more challenging tasks over several different sessions using the same module. ITSUS offers ultrasound anatomy training programs a unique opportunity to stratify content delivery to help learners at different skill levels spend time on appropriately challenging activities.

A systematic review of general SDL in health professions education found that self-directed modalities are associated with improved knowledge compared to traditional teaching methods. Advanced learners derive the most benefit from SDL [17]. SDL can nurture lifelong and independent learning skills vital to continued professional growth. This proctor-independent structure requires participants to troubleshoot scans, engage in dynamic interpretation of anatomy, encourage self-reliance, and enhance the skills taught by proctors during synchronous teaching. Giving users the flexibility to select their tasks further encourages students to take responsibility for their education and direct their learning toward individual needs.

By nature, in SDL, there is an absence of contemporaneous quality assurance of technique and image acquisition by a trained proctor. A study suggested that a complete lack of supervision in SDL can negatively affect knowledge development [18]. ITSUS addresses this by implementing faculty-guided quality assurance sessions and integration with a preexisting hands-on and didactic curriculum. Images accompany the guides to ensure that users practice proper techniques and understand the anatomy. This precisely designed and monitored approach to SDL allows for quality control of education.

A second potential disadvantage specific to self-directed, hands-on ultrasound is a lack of sufficient structure. ITSUS provides this structure by selecting fundamental anatomy and ultrasound tasks and providing detailed image acquisition and troubleshooting instructions. Although quality assurance cannot be performed simultaneously with image acquisition by participants, these scans can be reviewed by encouraging image portfolios, and feedback can be provided retrospectively.

A final advantage of the ITSUS curriculum is its potential use as a “flipped classroom.” The ITSUS curriculum can function in this way by providing constant access to educational resources that can then be applied in hands-on scanning sessions. Students can independently review the materials and instructions before engaging in a hands-on ultrasound practice session to derive maximum benefit. The “flipped classroom” model effectively improves deep and active learning [19]. ITSUS encourages students to engage deeply with their basic science knowledge and ultrasound skills by removing the proctor from scanning activities while promoting increased engagement and peer-to-peer interactions. In this model, structured, self-directed ultrasound provides an opportunity to encourage active and independent learning.

## Conclusions

The ITSUS program at the OSUCOM is a method for teaching anatomy and ultrasound in an asynchronous proctor-independent manner. This program can offset concerns about independent learning in general by providing a detailed structure and portfolio-building component. Compared to traditionally structured curricula, this method increases the flexibility of learning, encourages independent learning, improves access to resources, and requires fewer faculty hours. This is a model that can be added to an existing curriculum or can be embedded into curricula to enhance established programs.

## References

[1] Wittich CM, Montgomery SC, Neben MA (2002). Teaching cardiovascular anatomy to medical students by using a handheld ultrasound device. J Am Med Assoc.

[2] Tshibwabwa ET, Groves HM (2005). Integration of ultrasound in the education programme in anatomy. Med Educ.

[3] Tshibwabwa ET, Cannon J, Rice J, Kawooya MG, Sanii R, Mallin R (2016). Integrating ultrasound teaching into preclinical problem-based learning. J Clin Imaging Sci.

[4] Tshibwabwa ET, Groves HM, Levine MAH (2007). Teaching musculoskeletal ultrasound in the undergraduate medical curriculum. Med Educ.

[5] Syperda VA, Trivedi PN, Melo LC (2008). Ultrasonography in preclinical education: a pilot study. J Am Osteopath Assoc.

[6] Moore CL, Copel JA (2011). Point-of-care ultrasonography. N Engl J Med.

[7] Bahner DP, Goldman E, Way D, Royall NA, Liu YT (2014). The state of ultrasound education in U.S. medical schools: results of a national survey. Acad Med.

[8] (2021). AMA: privileging for ultrasound imaging H-230.960. https://policysearch.ama-assn.org/policyfinder/detail/Ultrasoundimaging?uri=%2FAMADoc%2FHOD.xml-0-1591.xml.

[9] Dinh VA, Fu JY, Lu S, Chiem A, Fox JC, Blaivas M (2016). Integration of ultrasound in medical education at United States medical schools: a national survey of directors' experiences. J Ultrasound Med.

[10] Kessler C, Bhandarkar S (2010). Ultrasound training for medical students and internal medicine residents--a needs assessment. J Clin Ultrasound.

[11] Prats MI, Royall NA, Panchal AR, Way DP, Bahner DP (2016). Outcomes of an advanced ultrasound elective: preparing medical students for residency and practice. J Ultrasound Med.

[12] Hoppmann RA, Rao VV, Bell F (2015). The evolution of an integrated ultrasound curriculum (iUSC) for medical students: 9-year experience. Crit Ultrasound J.

[13] Hoppmann RA, Rao VV, Poston MB (2011). An integrated ultrasound curriculum (iUSC) for medical students: 4-year experience. Crit Ultrasound J.

[14] Rao S, van Holsbeeck L, Musial JL (2008). A pilot study of comprehensive ultrasound education at the Wayne State University School of Medicine: a pioneer year review. J Ultrasound Med.

[15] Bahner DP, Adkins EJ, Hughes D, Barrie M, Boulger CT, Royall NA (2013). Integrated medical school ultrasound: development of an ultrasound vertical curriculum. Crit Ultrasound J.

[16] Bahner DP, Blickendorf JM, Bockbrader M (2016). Language of transducer manipulation. J Ultrasound Med.

[17] Murad MH, Coto-Yglesias F, Varkey P, Prokop LJ, Murad AL (2010). The effectiveness of self-directed learning in health professions education: a systematic review. Med Educ.

[18] Mahler SA, Wolcott CJ, Swoboda TK, Wang H, Arnold TC (2011). Techniques for teaching electrocardiogram interpretation: self-directed learning is less effective than a workshop or lecture. Med Educ.

[19] Mehta NB, Hull AL, Young JB, Stoller JK (2013). Just imagine: new paradigms for medical education. Acad Med.

